# Role of Platelet Parameters on Neovascular Glaucoma: A Retrospective Case-Control Study in China

**DOI:** 10.1371/journal.pone.0166893

**Published:** 2016-12-01

**Authors:** Shengjie Li, Wenjun Cao, Xinghuai Sun

**Affiliations:** 1 Department of Clinical Laboratory, Eye & ENT Hospital, Shanghai Medical College, Fudan University, China; 2 Department of Ophthalmology & Visual Science, Eye & ENT Hospital, Shanghai Medical College, Fudan University, China; 3 State Key Laboratory of Medical Neurobiology, Institutes of Brain Science, Fudan University, Shanghai, China; 4 Key Laboratory of Myopia, Ministry of Health (Fudan University), Shanghai, China; 5 Shanghai Key Laboratory of Visual Impairment and Restoration (Fudan University), Shanghai, China; Oregon Health and Science University, UNITED STATES

## Abstract

**Purpose:**

Retinal vein occlusion (RVO) and diabetic retinopathy (DR) are two major sight-threatening diseases which may lead to neovascular glaucoma (NVG). The aim of this study was to explore the association between platelet parameters and NVG.

**Methods:**

A total of 185 subjects were enrolled for the study from January, 2012 to December, 2015 at the Eye-ENT Hospital of Fudan University. Patients include those with NVG secondary to RVO (RVO group, n = 38), patients with NVG secondary to DR (DR group, n = 47), diabetics mellitus without retinopathy (DM group, n = 52), and healthy individuals (control group, n = 48). A complete ophthalmological examination including visual field examination, A-scan ultrasound, Fundus photography, and measurement of platelet parameters were performed for NVG subjects.

**Results:**

There was no statistical difference in the mean age and gender among the RVO, DR, and control groups (*p*>0.05). The mean level of platelet distribution width (PDW) was higher (*p*<0.001) in the RVO group (15.16±2.13fl) and DR group (16.17±1.66fl) when compared with the control group (13.77±2.99fl). The mean plateletcrit (PCT) value of the RVO group (0.229±0.063%) was also higher (*p* = 0.049) than the control group (0.199±0.045). In the DR group, mean platelet volume (MPV) value (10.72±1.57fl) was significantly higher (*p* = 0.002) than the control group (9.75±0.89fl). A similar trend was observed when platelet parameters were compared among the 3 groups with respect to age. The mean level of PDW was significantly higher (*p*<0.001) in the DR group (16.17±1.66fl) compared with the DM group (13.80±3.32fl). Stepwise multiple logistic regression analysis revealed that PDW (OR = 1.44, 95%CI = 1.149–1.805, *p* = 0.002) and MPV (OR = 1.503, 95%CI = 1.031–2.192, *p* = 0.034) were associated with the DR group, PDW (OR = 1.207, 95%CI = 1.010–1.443, *p* = 0.039) and PCT (OR = 1.663, 95%CI = 1.870–2.654, *p* = 0.036) were associated with the RVO group.

**Conclusion:**

Our results suggest that increased PDW and MPV are associated with the NVG secondary to DR group, elevated PDW and PCT are associated with the RVO group. It indicates that platelets might be an important factor in the onset and/or development of NVG.

## Introduction

Neovascular glaucoma (NVG) is a frequent complication associated with ischaemic retinopathies such as retinal vein occlusion (RVO) and diabetic retinopathy (DR). The disease process is characteristically refractory, difficult to treat and often results in vision loss [1–3]. The exact mechanism by which neovascularization and vision loss is inflicted on patients with RVO and DR remains unknown, but the three factors (stasis, vessel damage and hypercoagulability) involved in thrombogenesis, have been described in NVG patients [4–5].

Platelets play an important role in the pathogenesis of various thrombo-occlusive diseases, such as anterior ischemic optic neuropathy [6], ischemic and hemorrhagic stroke [7], and RVO [8]. Mean platelet volume (MPV) is a major indicator of the production rate and size of platelets, and has been associated with the activities of platelets [9, 10]. Large platelets are more reactive in metabolic and enzymatic activity than small platelets, and aggregate more easily than the latter [11, 12]. Platelet count reflects the production and aging of platelets, and is also an important platelet parameter [13, 14]. Two other platelet parameters are the plateletcrit (PCT) and the platelet distribution width (PDW), representing the fraction of platelets in blood and the variation in size of platelets, respectively [15, 16]. Yazgan S et al [17] suggested that the PCT and PDW were significantly higher in patients with PEX syndrome than in controls. Sahin A et al [8] reported that patients with RVO had significantly higher MPV values compared with the control subjects. Aksoy Y et al [18] also suggested that MPV values were significantly higher in branch RVO patients compared with the control subjects. However, inconsistent results showing that the MPV was significantly lower in patients with RVO than a control group were reported by Ornek N et al [19]. Moreover, to our knowledge, we did not find any articles that assessed platelet parameters in patients with NVG secondary to DR and RVO.

Several studies have shown that DR has higher MPV than healthy controls, which indicated that increased MPV may be a risk factor of retinopathy in DM patients [20, 21]. Citirik et al [22] reported that DR patients have a higher MPV than diabetic patients without DR. However, there are currently no reports on whether platelet parameters differ between DM patients and patients with NVG secondary to DR. Therefore, the aim of this study was to compare platelet parameters (platelet count, MPV, PCT and PDW) in patients with NVG with either RVO or DR, in comparison to DM and control subjects.

## Materials and Methods

### Patients

This was a retrospective, case-control study design. The study was approved by the Ethics Committee of the Eye-ENT Hospital of Fudan University, Shanghai, China and was conducted according to the Declaration of Helsinki. Written informed consent for the use of any clinical data in research was obtained for all patients at the time of admission to the Eye-ENT Hospital of Fudan University. Subjects, including those with NVG secondary to RVO and those secondary to DR, were recruited from the department of ophthalmology inpatient service at Eye-ENT Hospital of Fudan University from January 2012 to December 2015. Normal controls and DM patients were recruited from people through annual health screenings.

### Inclusion criteria

The diagnosis of NVG was made at the Eye-ENT Hospital of Fudan University. The definition of NVG was: (1) IOP>21 mmHg; (2) caused by retinal vascular disease (RVO or DR); (3) the presence of active neovascularization in the iris and/or angle; (4) with or without antiglaucomatous medications. [23] Newly diagnosed NVG patients and referral NVG patients were also included. Each patient underwent a standardized ophthalmic examination, which included refractive status, slit-lamp biomicroscopy, fundus examination, IOP (intraocular pressure), CCT (central corneal thickness), AL (axial length), ACD (anterior chamber depth), visual field examination, and gonioscopy, performed by glaucoma specialists. The MD and MS were measured by Octopus automated perimetry (HAAG, STREIT, Switzerland). A visual acuity measurement was obtained for each patient based on the International Standard Visual Acuity Chart. IOP was measured using the Goldmann applanation tonometry. Fundus photography was performed with a retinal camera (TRC-NW200, Topcon). A-scan ultrasound (A-Scan Pachymeter, Ultrasonic, Exton, PA, USA) was used to measure AL, ACD, and CCT. Patients with any systemic disease other than hypertension and diabetes mellitus were excluded from the study [8].

The NVG patients were divided into 2 categories for analysis: patients with open angle and high IOP (>21mmHg) due to neovascularization (O-NVG group); and patients with closed angle and high IOP (>21mmHg) (C-NVG group). [24, 25]

Normal controls had no ocular diseases, or systemic diseases such as diabetes, cardiovascular disease, anemia, autoimmune disease, cancer, and acute infectious disease. DM patients were excluded if they had any retinopathy or any other ocular diseases, as well as any systemic diseases such as cardiovascular disease, anemia, autoimmune disease, cancer, and acute infectious disease.

### Platelet parameters

Platelet parameters were measured with the Mindray BC-5500 (Shenzhen, China) automatic blood counting system. All blood samples in our study were collected in ethylenediaminetetraacetic acid (EDTA) tubes.

### Data analysis

The data were analyzed by SPSS13.0 (SPSS Inc., Chicago, IL). Results are presented as mean ± standard deviation (SD). Normality was assessed with the Kolmogorov-Smirnoff test. Chi-square test and Fisher exact tests were used for categorical variables. Baseline demographic information and clinical ocular parameters were compared between groups using the independent sample t test or one-way ANOVA test. The one-way ANOVA test was used to compare the levels of platelet parameters among the three groups. Multiple logistic regression analyses were performed to identify platelet risk factors associated with NVG patients with RVO or DR, compared to the control subjects. Odds ratios (ORs) with 95% confidence intervals (95% CIs) were estimated using logistic regression models. A P value of less than 0.05 was considered statistically significant.

## Results

### Characteristics of the study patients

A total of 38 NVG secondary to RVO patients (RVO group), 47 NVG secondary to DR patients (RVO group), 52 DM patients and 48 control subjects were enrolled in this study. Only one eye was selected randomly if both eyes suffered from NVG. The RVO group, DR group and control group were closely matched in terms of mean age and gender (*p* = 0.762, *p* = 0.736, respectively). No significant differences among the 3 groups were observed regarding any of the demographic and clinical ocular characteristics, except for diabetes mellitus (Table 1).

**Table 1:** Demographics of the study participants by NVG secondary to RVO patients, NVG secondary to DR patients, and controls.

	RVO group	DR group	Control group	t value	*p* value
Age (years)	56.32±15.41	54.09±10.75	55.08±15.23	0.272	0.762
Gender (male/female)	24/14	26/21	27/21	0.613	0.736
Diabetes mellitus	4 (10.53%)	47 (100%)	0 (0%)	142.116	<0.001
BMI, Kg/m^2^	23.99±3.71	22.72±3.47	24.80±4.19	1.818	0.171
Hypertension	22 (57.89%)	23 (48.94%)	20 (41.67%)	2.235	0.327
IOP (mmHg)	42.92±11.76	38.89±13.46	-	1.338	0.185
VCDR	0.65±0.24	0.59±0.19	-	0.899	0.375
MD (dB)	23.36±6.45	25.89±1.67	-	1.423	0.176
MS (dB)	4.51±6.76	1.78±1.45	-	1.478	0.161
Visual acuity	0.31±0.13	0.28±0.11	-	1.021	0.310

Data are expressed as mean± standard deviation (SD). Chi-square test, Fisher exact tests, independent sample t test and One-way ANOVA was used. BMI: body mass index, IOP: intraocular pressure, VCDR: vertical cup-disc ratio, MD: visual fields mean deviation, MS: visual fields mean sensitivity. RVO: neovascular glaucoma secondary to retinal vein occlusion. DR: neovascular glaucoma secondary to diabetic retinopathy.

### Comparison of PLT PDW, PCT, and MPV in RVO, DR, and control group

The mean level of PDW was significantly higher (*p*<0.001) in the RVO group (15.16±2.13fl) and the DR group (16.17±1.66fl) when compared with the control group (13.77±2.99fl). The mean PCT value of the RVO group (0.229±0.063%) was also significantly higher (*p* = 0.049) than the control group (0.199±0.045). In the DR group, MPV value (10.72±1.57fl) was significantly higher (*p* = 0.002) than the control group (9.75±0.89fl). There was no statistical difference in the PLT among the three groups (*p* = 0.108) (Table 2). RVO, DR, and control group were categorized as 3 subgroups (20–39 years, 40–59 years, and 60+ years) based on age. A similar trend was observed when PDW, PCT, and MPV were compared among the 3 subgroups (Table 3). However, among the RVO, DR, and control group aged 40–59 years, PDW level did not differ significantly (*p* = 0.208). Among patients aged 20–39 years and 60+ years, PCT level did not differ significantly (*p* = 0.484, *p* = 0.052, respectively). Among patients aged 40–59 years, the MPV level was not statistically different (*p* = 0.270).

**Table 2:** Laboratory findings that PLT, PDW, PCT, and MPV in RVO, DR, and control group.

	RVO group (n = 38)	DR group (n = 47)	Control group (n = 48)	t value	*p* value
PLT (10^9^/l)	228.71±72.11	205.43±60.55	202.06±53.08	2.267	0.108
PDW (fl)	15.16±2.13	16.17±1.66	13.77±2.99	12.533	<0.001^a^^,^^b^
PCT (%)	0.229±0.063	0.218±0.058	0.199±0.045	3.076	0.049^a^
MPV (fl)	10.20±1.44	10.72±1.57	9.75±0.89	6.297	0.002^b^

Data are expressed as mean± standard deviation (SD). One-way ANOVA was used. RVO: neovascular glaucoma secondary to retinal vein occlusion. DR: neovascular glaucoma secondary to diabetic retinopathy. MPV: mean platelet volume. PDW: platelet distribution width. PCT: plateletcrit. PLT: platelet count.

^a^P<0.05 for the difference between RVO group and Control group (1-way ANOVA with the LSD post hoc test).

^b^P<0.05 for the difference between DR group and Control group (1-way ANOVA with the LSD post hoc test).

^c^P<0.05 for the difference between RVO group and DR group (1-way ANOVA with the LSD post hoc test).

**Table 3:** Comparison of PDW, PCT, and MPV in RVO, DR, and control group, by age.

	RVO group (n = 38)	DR group (n = 47)	Control group (n = 48)	t value	*p* value
PDW (fl)					
20–39 years	14.13±3.06 (n = 6)	16.67±0.54 (n = 7)	14.12±2.98 (n = 12)	3.076	0.049^b^
40–59 years	15.50±1.71 (n = 16)	15.76±2.18 (n = 25)	14.49±2.80 (n = 16)	1.615	0.208
60+ years	15.21±2.13 (n = 16)	16.61±0.25 (n = 15)	12.99±3.10 (n = 20)	11.141	<0.001^a^^,^^b^
PCT (%)					
20–39 years	0.20±0.063 (n = 6)	0.23±0.052 (n = 7)	0.21±0.050 (n = 12)	0.749	0.484
40–59 years	0.244±0.043 (n = 16)	0.237±0.059 (n = 25)	0.203±0.036 (n = 16)	3.416	0.040^a^^,^^b^
60+ years	0.227±0.077 (n = 16)	0.179±0. 039 (n = 15)	0.189±0.049 (n = 20)	3.145	0.052
MPV (fl)					
20–39 years	9.78±1.05 (n = 6)	11.67±1.48 (n = 7)	10.03±0.82 (n = 12)	6.457	0.006^b^^,^^c^
40–59 years	10.22±1.37 (n = 16)	10.56±1.71 (n = 25)	9.81±0.89 (n = 16)	1.342	0.270
60+ years	10.34±1.67 (n = 16)	10.53±1.27 (n = 15)	9.55±0.93 (n = 20)	3.765	0.030^b^

Data are expressed as mean± standard deviation (SD). One-way ANOVA was used. RVO: neovascular glaucoma secondary to retinal vein occlusion. DR: neovascular glaucoma secondary to diabetic retinopathy. MPV: mean platelet volume. PDW: platelet distribution width. PCT: plateletcrit.

^a^P<0.05 for the difference between RVO group and Control group (1-way ANOVA with the LSD post hoc test).

^b^P<0.05 for the difference between DR group and Control group (1-way ANOVA with the LSD post hoc test).

^c^P<0.05 for the difference between RVO group and DR group (1-way ANOVA with the LSD post hoc test).

### Comparison of platelet parameters and demographics in O-NVG and C-NVG group

The NVG patients were divided into 2 categories for analysis: O-NVG group (n = 29) and C-NVG group (n = 56). Because the NVG was defined as IOP >21 mmHg in this study, patients with simply iris neovascularization (without IOP elevation) was lacking. No significant differences between the O-NVG group and C-NVG group were observed in terms of the demographic and platelet parameters, except for hypertension (Table 4). The C-NVG group has a higher level of VCDR and MD than the O-NVG group (p = 0.005, p = 0.005, respectively). Moreover, the level of MS and visual acuity was higher in the O-NVG group than the C-NVG group (p = 0.010, p = 0.014, respectively).

**Table 4:** Comparison of platelet parameters and demographics in O-NVG and C-NVG group.

	O-NVG group (n = 29)	C-NVG group (n = 56)	t value	*p* value
Age (years)	54.17±11.15	55.55±13.92	0.462	0.645
Gender (male/female)	17/12	33/23	0.001	0.978
Diabetes mellitus	17 (58.62%)	34 (60.715)	0.035	0.852
Hypertension	5 (17.24%)	40 (71.43%)	22.518	<0.001
BMI, Kg/m^2^	22.51±3.27	22.02±7.25	0.279	0.781
PLT (10^9^/l)	213.86±70.06	216.86±65.34	0.195	0.845
PDW (fl)	15.59±2.04	15.79±1.89	0.433	0.666
PCT (%)	0.221±0.057	0.224±0.062	0.222	0.825
MPV (fl)	10.65±1.73	10.40±1.42	0.704	0.483
IOP (mmHg)	43.41±10.39	41.11±14.62	0.749	0.456
VCDR	0.51±0.17	0.70±0.20	3.004	0.005
MD (dB)	20.78±5.87	27.04±0.94	3.508	0.005
MS (dB)	6.90±6.36	0.77±1.00	3.174	0.010
Visual acuity	0.33±0.11	0.27±0.12	2.521	0.014

Data are expressed as mean± standard deviation (SD). Chi-square test and independent sample t test was used. O-NVG: open angle and high IOP (>21mmHg) due to neovascularization, C-NVG: closed angle and high IOP (>21mmHg) due to neovascularization, BMI: body mass index, IOP: intraocular pressure, VCDR: vertical cup-disc ratio, MD: visual fields mean deviation, MS: visual fields mean sensitivity.MPV: mean platelet volume. PDW: platelet distribution width. PCT: plateletcrit. PLT: platelet count.

### Comparison of PLT PDW, PCT, and MPV in DM, DR, and control group

No significant differences among the DM (diabetics mellitus without retinopathy), DR (patients with NVG secondary to DR), and control groups were observed regarding any of the demographic (Table 5). The DM group, DR group, and control group were closely matched in terms of mean age and gender. The mean level of PDW was significantly higher (*p*<0.001) in the DR group (16.17±1.66fl) compared to the DM group (13.80±3.32fl) and the control group (13.77±2.99fl). However, PDW level did not differ significantly between the DM group and control group. The mean level of MPV was significantly higher (p<0.001) in the DM group (10.39±0.90fl) and DR group (10.72±1.57fl) compared to the control group (9.75±0.89fl). The DR group has a higher level of MPV than the DM group, but this was not statistically different. There was no statistical difference in the PLT and PCT among the three groups (Table 5).

**Table 5:** Laboratory findings that PLT, PDW, PCT, and MPV in DM, DR, and control group.

	DM group	DR group	Control group	t value	*p* value
Age (years)	55.08±15.23	54.09±10.75	55.08±15.23	0.077	0.926
Gender (male/female)	31/21	26/21	27/21	0.209	0.901
Diabetes mellitus	52 (100%)	47 (100%)	0 (0%)	147.00	<0.001
Hypertension	25 (48.08%)	23 (48.94%)	20 (41.67%)	0.612	0.736
PLT (10^9^/l)	205.33±86.02	205.43±60.55	202.06±53.08	0.038	0.963
PDW (fl)	13.80±3.32	16.17±1.66	13.77±2.99	11.786	<0.001^b^^,^ ^c^
PCT (%)	0.214±0.066	0.218±0.058	0.199±0.045	1.630	0.200
MPV (fl)	10.39±0.90	10.72±1.57	9.75±0.89	8.433	<0.001^a^^,^ ^b^

Data are expressed as mean± standard deviation (SD). One-way ANOVA was used. DM: diabetes mellituswithout retinopathy. DR: neovascular glaucoma secondary to diabetic retinopathy. MPV: mean platelet volume. PDW: platelet distribution width. PCT: plateletcrit. PLT: platelet count.

^a^P<0.05 for the difference between DM group and Control group (1-way ANOVA with the LSD post hoc test).

^b^P<0.05 for the difference between DR group and Control group (1-way ANOVA with the LSD post hoc test).

^c^P<0.05 for the difference between DM group and DR group (1-way ANOVA with the LSD post hoc test).

### The association of PDW, PCT, and MPV with RVO, DR, and control individuals by multiple logistic regression analysis

Stepwise multiple logistic regression analysis revealed that PDW (OR = 1.44, 95%CI = 1.149–1.805, *p* = 0.002) and MPV (OR = 1.503, 95%CI = 1.031–2.192, *p* = 0.034) were associated with DR after adjusting for age, sex, PLT, PDW, PCT, MPV, and hypertension (Table 6). PDW (OR = 1.207, 95%CI = 1.010–1.443, *p* = 0.039) and PCT (OR = 1.663, 95%CI = 1.870–2.654, *p* = 0.036) were associated with RVO after adjusting for age, sex, PLT, PDW, PCT, MPV, and hypertension (Table 7).

**Table 6:** Multiple logistic regression analysis of association of PDW and MPV with NVG secondary to DR patients and in control individuals.

Risk factors	Odds Ratio	95% Confidence Interval	*p* value ^a^
PDW (fl)	1.44	1.149–1.805	0.002
MPV (fl)	1.503	1.031–2.192	0.034

^a^ Adjusted for age, sex, platelet count, platelet distribution width, plateletcrit, mean platelet volume, hypertension. MPV: mean platelet volume. PDW: platelet distribution width.

**Table 7:** Multiple logistic regression analysis of association of PDW and PCT with NVG secondary to RVO patients and in control individuals.

Risk factors	Odds Ratio	95% Confidence Interval	*p* value ^a^
PDW (fl)	1.207	1.010–1.443	0.039
PCT (%)	1.663	1.870–2.654	0.036

^a^ Adjusted for age, sex, platelet count, platelet distribution width, plateletcrit, mean platelet volume, hypertension. PDW: platelet distribution width. PCT: plateletcrit

## Discussion

Retinal ischemia and macular oedema arising from RVO and DR are the most common cause of vision loss [26]. The exact mechanism of NVG secondary to RVO or DR is multifactorial and remains unknown. It has been shown that platelets play an important role in the pathophysiology of retinal artery occlusion [27], nonarteritic anterior ischemic optic neuropathy [6], ischemic and hemorrhagic stroke [7], and cardiovascular diseases [28]. In this study, 38 patients with NVG secondary to RVO and 47 patients with NVG secondary to DR were studied with regard to platelet parameters and compared to control subjects. Moreover, 47 patients with NVG secondary to DR were studied with regard to platelet parameters and compared to DM patients without retinopathy.

The main finding in the present study was that PDW and PCT were higher in patients with NVG secondary to RVO than in the control group and associated with higher prevalence of NVG secondary to RVO according to multiple logistic regression analysis. Furthermore, the levels of PDW and MPV in patients in the NVG secondary to DR group were higher than in the control group. Multiple logistic regression analysis revealed a significant association between PDW and MPV with NVG secondary to DR. The above association remained significant even after adjustment for age, sex, PLT, PDW, PCT, MPV, and hypertension. Our results suggest that platelet function might be an important factor in the onset and/or development of NVG. Furthermore, we found that the mean level of PDW was significantly higher in the NVG secondary to DR group compared with the DM group.

There has been a few studies which have evaluated the relationship between MPV and cardiovascular disease [27, 29–31]. Sahin M et al [27] reported that patients with retinal artery occlusion had significantly higher MPV values compared with control subjects and was an independent predictor of retinal artery occlusion. MPV as risk factors has been studied in patients with deep vein thrombosis. Han JS et al [31] reported that median MPV was higher in deep vein thrombosis patients and could be considered as a meaningful laboratory marker for deep vein thrombosis. In addition, MPV is elevated in sinus thrombosis [32] and pulmonary thromboembolism [33].These studies suggest that a higher MPV was a risk factor for thrombogenesis.

In our study, the MPV values were significantly higher in patients with NVG secondary to DR and associated with higher prevalence of NVG secondary to DR according to multiple logistic regression analysis. Citiriket al [22] also found that DR patients have increased MPV values compared with healthy subjects, and similar results were reported by another study [34]. Therefore, a higher MPV seems to be a risk factor for DR. In our study, the MPV values in RVO patients were also higher than in the control group but not statistically significant. However, a few studies reported that the MPV values were significantly higher in patients with RVO [18, 35], and inconsistent results were also reported where the MPV was significantly lower in patients with RVO than the control group [19]. Therefore, we expect future studies to confirm the relationship between MPV and patients with NVG secondary to RVO.

PDW is another important parameter reflecting platelet function. PDW represents the variation in size of platelets and PDW has been investigated as a marker of platelet activation [16, 36]. A higher PDW has been previously observed in patients with coronary artery disease and myeloproliferative disorders [37, 38]. We found that PDW were higher in patients with NVG secondary to RVO and DR than in the control group and associated with higher prevalence of NVG according to multiple logistic regression analysis. PDW was also significantly increased in cerebral venous sinus thrombosis and might be associated with the severity of cerebral venous sinus thrombosis [32]. To our knowledge, there was only one study that explored the relationship between PDW and DR, but DR patients have no different PDW values compared with healthy subjects [22]. No study has examined the relationship between PDW and patients with NVG secondary to RVO. Therefore, this is the first study to report that PDW was higher in patients with NVG secondary to RVO and DR and suggests that increased PDW may be related to NVG.

PCT is the percentage of platelet mass in the blood and is regarded as an indicator of circulating platelets in a unit volume of blood [34]. Several studies have reported that a higher PCT level was associated with cardiovascular disease such as slow coronary flow [39], coronary artery disease [40, 41]. However, no study has investigated the relationship between PCT and NVG secondary to RVO. Akpinar I et al [39] reported that PCT level was higher in slow coronary flow patients than those without slow coronary flow. Ugur M et al [40] found that with high PCT values, cardiovascular patients had a worse prognosis. In the present study, PCT value in patients with NVG secondary to RVO was significantly higher than the control group. In addition, PCT was a risk factor of NVG secondary to RVO on the basis of a multiple logistic regression analysis adjusted for age, sex, PLT, PDW, MPV, and hypertension. This suggests that a higher PCT might be a risk factor of thrombogenesis in patients with NVG secondary to RVO.

In our study, we found that the mean level of PDW was significantly higher in patients with NVG secondary to DR group compared with the DM group. MPV was also higher in the NVG secondary to DR group than the DM group, although this difference was not statistically significant. PDW and MPV are increased during platelet activation, and this can increase the chance of vascular complications [41]. Papanas et al [42] reported that patients with DR have higher MPV levels compared with other diabetic patients. Jindal et al [16] showed that PDW levels were significantly higher in diabetic patients and the level of PDW is increased more significantly in patients with microvascular complications. However, Citirik et al [22] found that DM patients have significantly higher MPV and PDW values compared to healthy subjects, but MPV and PDW levels were not altered along with the DR stage. We thought that the following factors might explain this observation: (1) the elapsed time of MPV measurement may be different, in this study the blood was studied within 30 minutes; (2) the subjects of the above studies differed from our study, this study investigated NVG secondary to DR, whereas the above study explored DR or DM patients only. To the best of our knowledge, this is the first study to explore whether platelet parameters differ between DM and NVG (secondary to DR).

Our study did have some limitations. (1) The sample size is relatively small. A number of patients were subsequently excluded due to the strict inclusion criteria; and to our knowledge, this is the first study examining platelet parameters within NVG secondary to RVO and DR patients. (2) Our study was a single-center, retrospective analysis. The results might be affected by confounding factors, despite a multiple logistic regression analysis was performed to adjust for age, sex, PLT, PDW, MPV, and hypertension. Therefore, larger-scale, multi-center prospective studies are required to better investigate the relationship between platelets with RVO and DR patients.

In conclusion, our results suggested that increased PDW and MPV are associated with NVG secondary to DR, and elevated PDW and PCT increases the risk for NVG secondary to ROV. Moreover, the mean level of PDW was significantly higher in the NVG secondary to DR group when compared with the DM group. Platelets may not be the primary cause of NVG with RVO or DR but may be a secondary factor that could increase the prevalence of NVG, because platelet parameters values were still within the reference range.
